# Evaluating the Acceptability and Feasibility of a Sexual Health–Focused Contraceptive Decision Aid for Diverse Young Adults: User-Centered Usability Study

**DOI:** 10.2196/44170

**Published:** 2023-10-03

**Authors:** Rose Goueth, Blair Darney, Aubri Hoffman, Karen B Eden

**Affiliations:** 1 Department of Medical Informatics and Clinical Epidemiology Oregon Health & Science University Portland, OR United States; 2 Department of Obstetrics and Gynecology Oregon Health & Science University Portland, OR United States; 3 Portland State University School of Public Health Oregon Health & Science University Portland, OR United States; 4 Centro de Investigacion en Salud Poblacional (CISP) Insituto Nacional de Salud Publica (INSP) Cuernavaca Mexico; 5 Value Institute for Health and Care Dell Medical School The University of Texas at Austin Austin, TX United States; 6 Pacific Northwest Evidence-Based Practice Center Department of Medical Informatics and Clinical Epidemiology Oregon Health & Science University Portland, OR United States

**Keywords:** decision aid, contraception, decision-making, user-centered design, young adults, pilot study, feasibility, acceptability, development, support, tool, survey, sexual health

## Abstract

**Background:**

Young adults with low sexual health literacy levels may find it difficult to make informed decisions about contraceptive methods. We developed and pilot-tested a web-based decision aid—Healthy Sex Choices—designed to support diverse young adults with their contraceptive decision-making.

**Objective:**

This pilot study aimed to evaluate whether the Healthy Sex Choices decision aid is acceptable and feasible to patients and clinicians.

**Methods:**

We used the Ottawa Decision Support Framework and the International Patient Decision Aid Standards to develop and pilot the decision tool. We first conducted a needs assessment with our advisory panel (5 clinicians and 2 patients) that informed decision aid development. All panelists participated in semistructured interviews about their experience with contraceptive counseling. Clinicians also completed a focus group session centered around the development of sex education content for the tool. Before commencing the pilot study, 5 participants from ResearchMatch (Vanderbilt University Medical Center) assessed the tool and suggested improvements.

**Results:**

Participants were satisfied with the tool, rating the acceptability as “good.” Interviewees revealed that the tool made contraceptive decision-making easier and would recommend the tool to a family member or friend. Participants had a nonsignificant change in knowledge scores (53% before vs 45% after; *P*=.99). Overall, decisional conflict scores significantly decreased (16.1 before vs 2.8 after; *P*<.001) with the informed subscale (patients feeling more informed) having the greatest decline (23.1 vs 4.7; mean difference 19.0, SD 27.1). Subanalyses of contraceptive knowledge and decisional conflict illustrated that participants of color had lower knowledge scores (48% vs 55%) and higher decisional conflict (20.0 vs 14.5) at baseline than their white counterparts.

**Conclusions:**

Participants found Healthy Sex Choices to be acceptable and reported reduced decisional conflict after using the tool. The development and pilot phases of this study provided a foundation for creating reproductive health decision aids that acknowledge and provide guidance for diverse patient populations.

## Introduction

Access to and information about reproductive health care in the United States has changed rapidly over the last decade [1-3]. Many people rely on informal information sources (eg, personal social networks and the Internet) and receive information that can be inaccurate, not culturally relevant, and not written in plain language [4,5]. Previous studies have also established that young people have low sexual health literacy levels, regardless of age, sex, race, ethnicity, or educational level [6]. Patients can access sexual health information and related services in a clinical setting. Clinicians are trained to perform patient-centered contraceptive counseling to ensure patients’ final contraceptive option aligns with their preferences and values [7]. Effective counseling can impact health outcomes (ie, contraceptive use and unintended pregnancy) yet clinicians face several barriers (eg, time constraints and patient gaps in sexual health knowledge) that pose challenges to engaging in contraceptive counseling [8-11].

Patient decision aids—evidence-based tools designed to form realistic expectations, educate patients about treatment options with plain language, and guide them through decision-making—provide a solution to many of the current barriers clinicians face in providing care [12]. More specifically, technology-based decision aids provide patients with an accessible tool to use at their own time and pace, outside of the clinical environment. Patients who use decision aids appear to increase contraceptive use, knowledge, and contraceptive satisfaction while reducing decisional conflict [13,14]. What is missing from the current decision aid landscape is one that (1) is intentionally built to be inclusive for underserved populations (ie, racial or ethnic and gender nonbinary people), groups who experience worse reproductive health outcomes than their White or heterosexual counterparts [11,15,16], and (2) integrates sexual health data into the electronic health record for use at current and future health care visits. We designed a web-based sexual health–focused contraceptive decision aid for young adults (18-24 years old) from diverse populations with a user-centered design approach and decision science framework and standards. Our objective was to evaluate the acceptability and feasibility of the Healthy Sex Choices decision aid for patients.

## Methods

### Intervention Development

**Figure 1:**
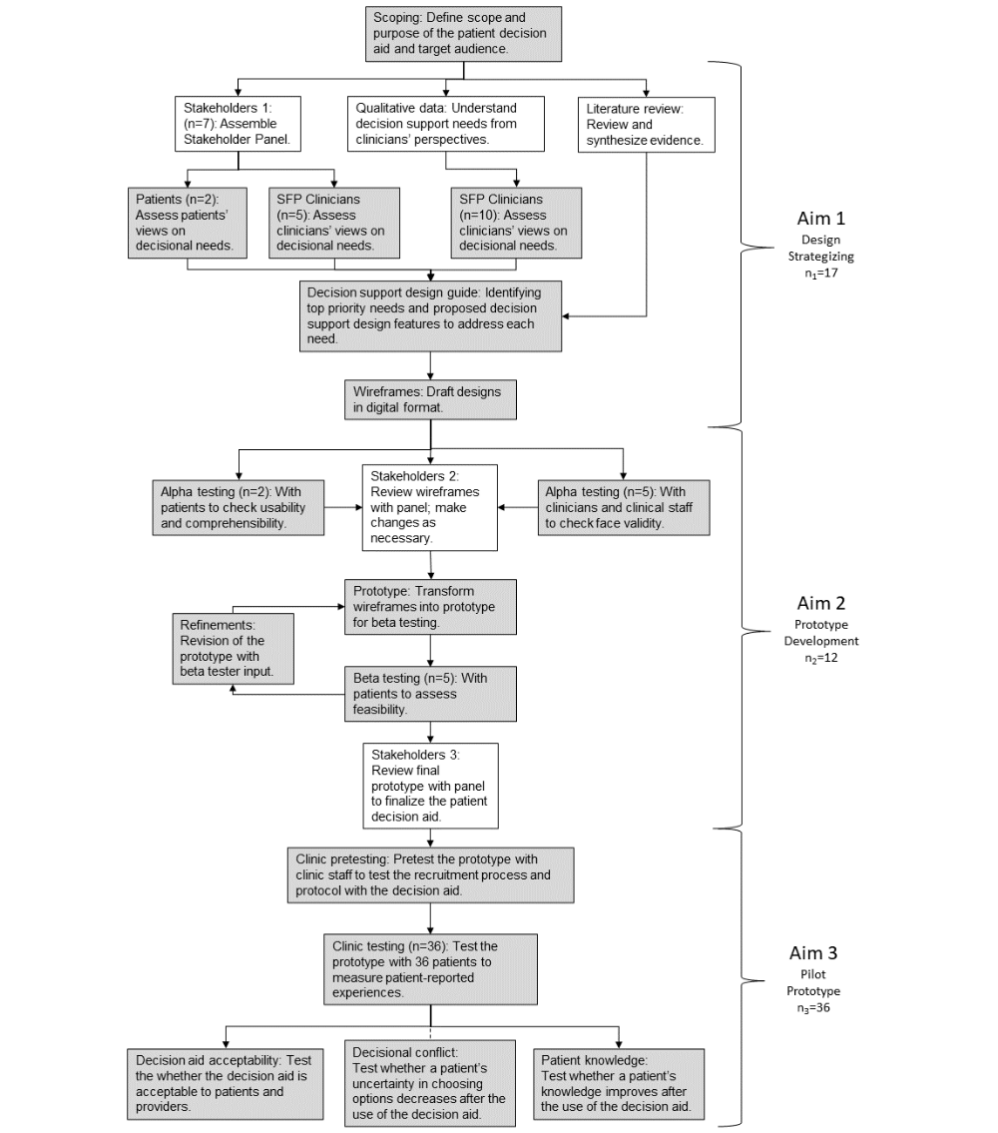
The model development process (adapted for this study) [19]. SFP: society of family planning.

We used user-centered design principles, the Ottawa Decision Support Framework, and the International Patient Decision Aid Standards (IPDAS) to develop this intervention. The Ottawa Decision Support Framework postulates that decision-support interventions that address decisional needs will improve decision-making and decision quality [17]. IPDAS are quality standards and processes that aid in the development and implementation of patient decision aids [18]. We adapted IPDAS’s Development Process Model (Figure 1 [19]) to outline the steps needed to develop and refine the Healthy Sex Choices tool. We assembled an advisory panel made up of 5 clinicians from the Society of Family Planning (a multidisciplinary research community of those engaged in the science and medicine of abortion and contraception) and 2 patients to engage in our needs assessment, informing the development process of the decision aid. All panelists participated in semistructured interviews about their experiences conducting or participating in contraceptive counseling. Clinicians also shared discourse around potential solutions to challenges in contraceptive counseling, how decision aids can ameliorate barriers to care, and how we can implement suggested solutions with a health equity lens.

**Figure 2:**
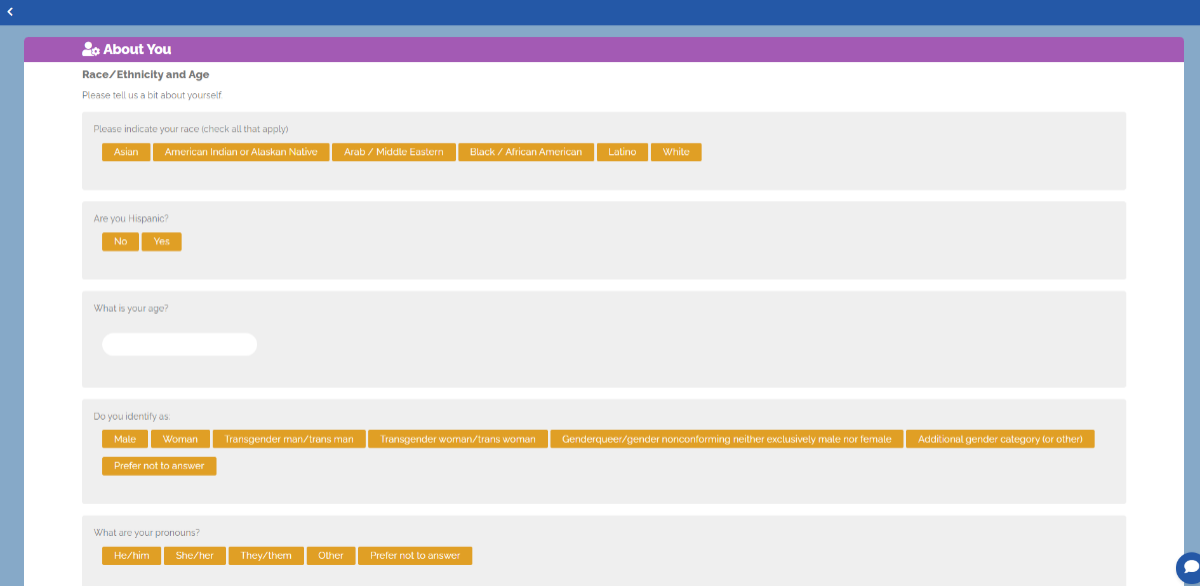
The Healthy Sex Choices decision aid patient demographic page.

**Figure 3:**
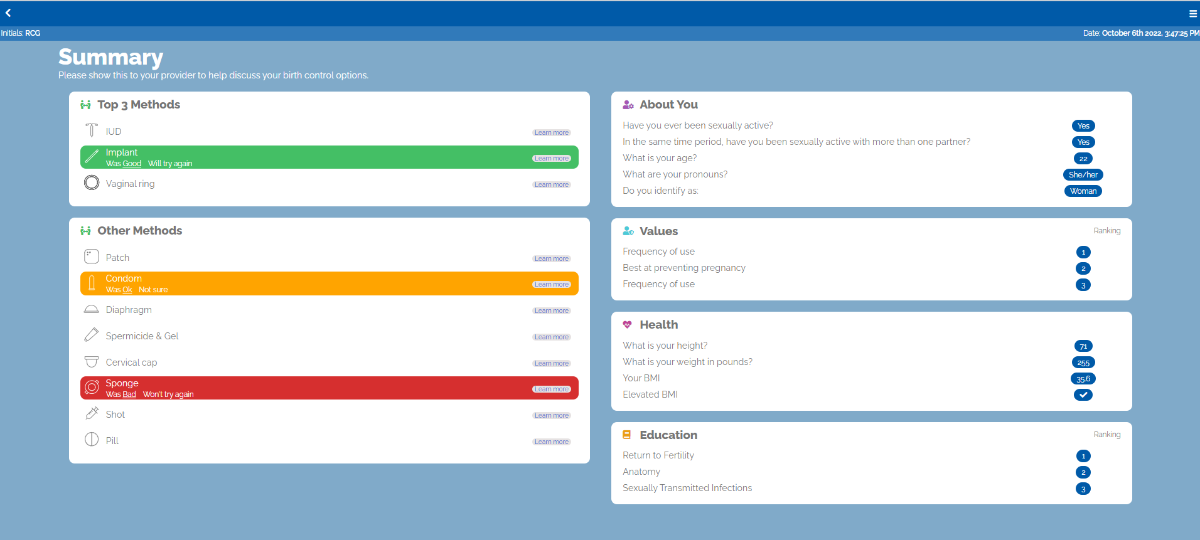
The Healthy Sex Choices decision aid summary page.

Clinician interview data revealed the need to customize patient care to address and acknowledge identity complexity [20]. The solution materialized (through conversations with advisory panel members and the decision aid developer) with the About Me module. Patients to identify their race or ethnicity, gender identity, sexual orientation, and pronouns within the module, creating a safe space for patients to share their identities with clinical staff (Figure 2) and providing clinicians with clear information on how to address and advise patients during visits through the decision aid summary page (Figure 3).

Advisory panel clinicians also participated in a focus group session centered around crafting sex education pieces related to contraceptive choice. These participants identified seven education topics: (1) female anatomy; (2) return to fertility; (3) sexual wellness; (4) consent, coercion, and violence; (5) contraceptive side effects; (6) sexually transmitted infections; and (7) emergency birth control. We refined these topics using content from Planned Parenthood.

Once we constructed a web-based prototype of the decision aid, we asked the advisory panel for feedback and further refined the tool before testing with 5 participants (participant characteristics found in Table 1) recruited from ResearchMatch—a recruitment volunteer database used for clinical and health-related research studies [21]. During beta-testing, we learned that participants found the tool to be easy to read, had a nice organization, and was not overwhelming to use. Testers suggested minor changes including misspellings and correcting the functionality of the summary page.

**Table 1:** Beta-testing participant characteristics (N=5).

Characteristic			Value
Age (years), mean (IQR)			21 (18-22)
**Race or ethnicity, n (%)**			
	White (non-Hispanic)	4 (80)	
	Multirace (non-Hispanic)	1 (20)	
**Gender identity, n (%)**			
	Female	5 (100)	
**Patient census region, n (%)**			
	Midwest	3 (60)	
	Northwest	1 (20)	
	South	0 (0)	
	West	1 (20)	

### Intervention

**Figure 4:**
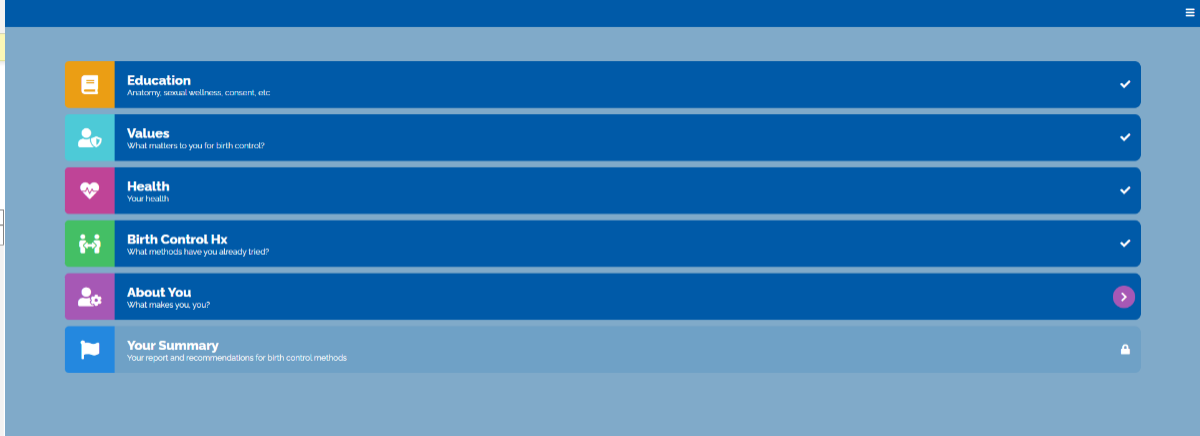
The Healthy Sex Choices decision aid main page.

**Figure 5:**
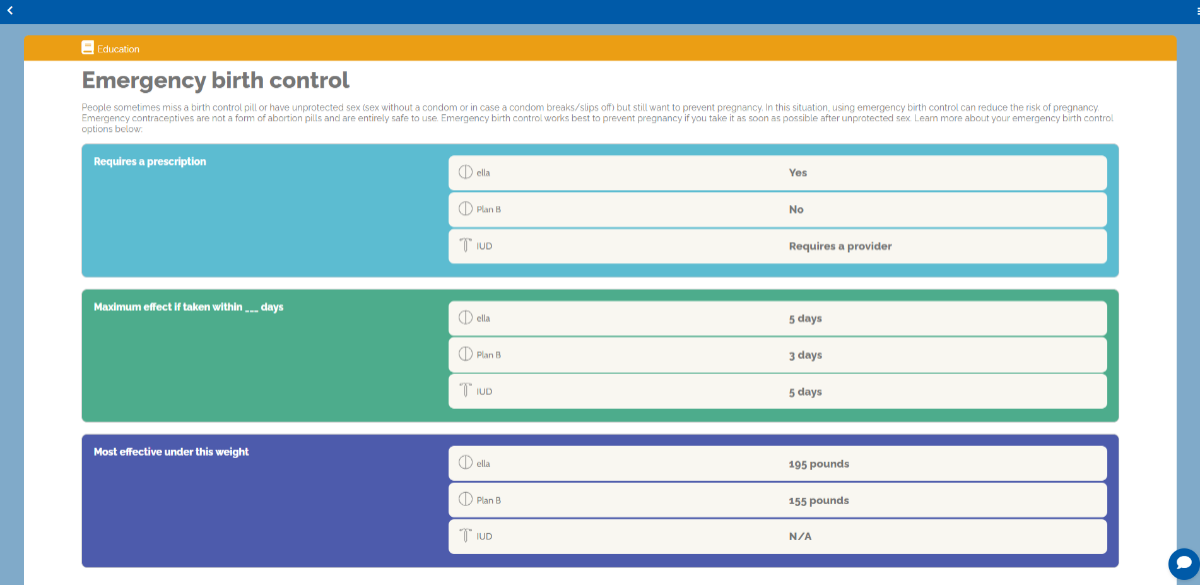
The Healthy Sex Choices decision aid education page.

The web-based Healthy Sex Choices tool starts with an educational module covering topics deemed important through the needs assessment with clinicians, described above (Figures 2-5). Patients were able to bookmark educational pieces they wanted to discuss with their clinician by clicking yes or no next to the, “Would you like to discuss this topic with your provider?” question at the bottom of every education content page (Figure 5). Once the user finished the education module, they were asked to rank their bookmarked education topics in order of importance. Then participants went through an assessment comprised of 3 modules (Values, Health, and Birth Control History) that asked about the importance of contraceptive attitudes and values including previously tried methods and current sexual activity. Patients then completed a fourth module (About Me) about their identities (ie, race or ethnic background, sexual orientation, gender identity, and pronouns) to support patient-clinician communication and acknowledge patients’ identities during their visit (Figure 2). The final module (Your Summary) recommends contraceptive options that best map to the patients’ attitudes and values and allows them to rank the recommended methods in order of preference. When the patient completes the application, they receive a summary page with links to explore each contraceptive option on the Planned Parenthood website (Figure 3).

### Ethics Approval

Oregon Health & Science University’s (OHSU) institutional review board (00022943) reviewed and approved all study activities. We provided US $35 gift cards to participants who completed all advisory board tasks. Participants received a US $20 gift card for completing all pilot study tasks and those who additionally completed a 15-minute interview about their experience using the tool received a US $35 gift card. To preserve privacy and confidentiality, we deidentified data used for this research and preserved it on an encrypted OHSU-owned hard drive within OHSU-firewalled cloud storage.

### Study Design

We conducted a nonrandomized before and after study of the Healthy Sex Choices decision aid in a Planned Parenthood Columbia Willamette clinic between May and July 2022. We recruited participants to evaluate the acceptability and feasibility of the tool and to assess its implementation for future work.

### Recruitment

We recruited patients ages 18-24 years who were coming in for a contraceptive-related visit to discuss contraceptive options; were not currently pregnant; were able to become pregnant; had the ability to read, speak, and write in English; and had access to a laptop or mobile phone connected to the Internet at the time of the visit. We utilized front office staff and flyers posted throughout the clinic for recruitment. Participants could enroll in the study via a QR code on the flyer. The office manager identified eligible patients when reviewing patient appointments each morning. The front office staff then asked identified patients if they wanted to participate in the study. We started recruitment with a narrow purposive sample to obtain at least 20% of persons of color within our sample, afterward, all patients were eligible for recruitment.

Once patients agreed to the web-based informed consent form via Research Electronic Data Capture (REDCap; Vanderbilt University), they completed a REDCap before-intervention survey, used the decision aid before seeing their clinician, and reviewed the summary given by the tool during the visit with the clinician. Immediately after the visit, patients filled out a postintervention survey. They received email reminders each day for up to 3 days to complete the survey.

### Outcome Measures

#### Acceptability

We assessed acceptability using an adapted version of the Ottawa Acceptability Scale, a 10-point questionnaire with scaled and free-text responses after the visit [22]. The 10-item scale measures patients’ comprehensibility of the decision aid’s components and its overall suitability for decision-making [22]. More specifically, we assessed scaled questions through averages and summarized free-text responses. We also conducted patient interviews to further understand refinements to improve the use of the tool.

#### Patient Knowledge

We assessed knowledge before and after using the intervention using an adapted version of the Contraceptive Knowledge Assessment [23]. Our version contained 15 questions mapping to several educational pieces given within the tool covering (1) female anatomy, (2) return to fertility, (3) contraceptive side effects, (4) sexually transmitted infections, and (5) emergency birth control. Each multiple-choice assessment question contained 5 options and scored using percent correct.

#### Decisional Conflict

Decisional conflict was measured before and after using the decision aid using the 10-item, Low Literacy version of the Decisional Conflict Scale [24,25]. The 10-item version measures patients’ perceptions of their uncertainty in choosing options, factors attributing to the uncertainty, and the ability to perform effective decision-making. The scale grades each question on a 0, 2, and 4 scale, where 0=yes, 2=unsure, and 4=no. The final score ranges from 0=no decisional conflict to 100=extremely high decisional conflict.

### Sample Size Calculation

A formal sample size calculation would not be appropriate for this pilot study; however, samples of 20-30 participants are consistent with pilot study decision aid literature [26,27]. Previous studies also report that 20-25 participants yield effect sizes of 5%-10% increases in Knowledge and Decisional Conflict Scale scores, our secondary and exploratory measures [26,27]. A sample size of 31 should detect a 10% difference in patient knowledge and decisional conflict, with an α of .05 and a power of 80%. To account for a 10% noncompletion rate, we planned to screen and recruit at least 36 patients.

### Statistical Analyses

We used paired 2-tailed *t* tests to compare patient knowledge and decisional conflict before and after the intervention and used the SD to assess differences within the subscales rather than *P* values to account for intercorrelation. We then conducted an exploratory analysis to assess the differences between race or ethnicity groups with SD and summary statistics. We averaged quantitative data from the acceptability scale and website analytic data (ie, decision aid completion times). We used a *P* value of .05 or less to determine the significance of statistical tests.

## Results

### Participant Characteristics

Of the 70 young people who initiated the study, 67 were eligible and 31 enrolled but did not complete participation in the study. A total of 36 participants consented and completed the study. Over one-third of participants identified as a person of color 36% (13/36). The average age was 22 years and most participants identified as women 94% (34/36). Participants primarily lived in Oregon (44%, 14/36) and Washington State (39%, 16/36) of the Pacific Northwest region and one participant lived in California (3%, 1/36; Table 2).

**Table 2:** Pilot study participant characteristics (N=36).

Characteristic		Value
Age (years), mean (IQR)		22 (18-24)
**Age group (years), n (%)**		
	18-19	8 (25)
	20-22	15 (42)
	23-24	13 (36)
**Race or ethnicity, n (%)**		
	Black or African American	3 (8)
	Hispanic or Latino	6 (17)
	Native Hawaiian or other Pacific Islander	1 (3)
	White	22 (61)
	Multiracial	3 (8)
	Refuse to answer	1 (3)
**Gender identity, n (%)**		
	Female	34 (94)
	Nonbinary	2 (6)
**Education level, n (%)**		
	Did not graduate high school	2 (6)
	High school diploma or GED	9 (25)
	Some college	14 (39)
	Associate degree	3 (8)
	Bachelor degree	8 (22)
**Current marital status, n (%)**		
	Never married	25 (69)
	Living with a partner or significant other	11 (31)
**State of residence, n (%)**		
	California	1 (3)
	Oregon	16 (44)
	Washington	14 (39)
	Not Reported	5 (14)

### Outcomes

Most participants were satisfied with the tool and rated its acceptability as “good.” More specifically, patients found the tool to be the right length 89% (32/36), had enough information 86% (31/36), and a balanced presentation 72% (26/36) and all found the tool to be useful when choosing a contraceptive 100% (36/36). Participants reported the tool made it easier to choose a contraceptive option and would recommend the tool to a friend or family member to use the tool before a clinical visit. The average tool completion time was 8 minutes.

There was a nonsignificant change in patient knowledge scores (percent correct) after using the decision aid (53% vs 45%; *P*=.99; Table 3). Total decisional conflict scores significantly decreased after using the intervention (16.1 vs 2.78; *P*<.001). In all 4 decisional conflict subscales, we saw a decrease after using the decision aid, with the greatest decrease in the informed subscale (23.1 vs 4.7, mean difference 19.0, SD 27.1).

**Table 3:** Before or after intervention outcome changes.

Outcomes			Before intervention, mean		After intervention, mean		Mean difference (SD)		*P* value
Patient knowledge			53%		45%		7.1		.99
**Decisional conflict**			16.1		2.8		13.3		<.001
	Uncertainty subscore	20.8		4.2		16.7 (28.0)			
	Informed subscore	23.1		4.2		19.0 (27.1)			
	Values clarity subscore	12.5		2.1		10.4 (25.6)			
	Support subscore	8.33		0.9		7.4 (14.1)			

### Exploratory Analysis

Our exploratory subgroup analyses for race or ethnicity (n=35) revealed that on average, participants of color had lower knowledge scores (48% vs 55%) and higher decisional conflict (20.0 vs 14.5) at baseline than their white counterparts. Yet, participants of color had a larger decrease in overall decisional conflict (15.8 mean difference vs 12.5 among White participants) and greater decreases in values clarity and support subscales. White patients had a greater significant decrease in the informed subscale.

## Discussion

### Principal Results

The development of the Health Sex Choices tool used patient-centered and health equity approaches to provide patients with a foundation of sex education knowledge and aid patients with their contraceptive choice through an assessment. In evaluating the tool with a racially and ethnically diverse population, patients found the tool to be acceptable overall, and the tool reduced overall decisional conflict by 82% but generated no significant change in patient knowledge. Our exploratory analysis revealed patients of color had lower baseline knowledge and higher decisional conflict but had a larger decrease in decisional conflict after using the intervention compared to their white counterparts.

Decision aids provide the ability for patients to learn and explore treatment options in a safe space and perform decision-making tasks that acknowledge their values and preferences. These aids provide a common language and guide for a shared decision-making session. The novelty of this research was to provide a decision aid that not only acknowledges patients’ identities, preferences, and values, but also their intersectionality, as it plays a big part in young adults’ decision-making, receipt of care, and the overall improvement of health equity [28,29].

### Comparison With Prior Work

Some results in this study mapped to results of recent systematic reviews; one that quantified the effects of the use of technology-based contraceptive decision aids and another that evaluated decision aids used in obstetrics and gynecology more broadly [13,14]. Both systematic reviews reported higher decision self-efficacy scores and lower decisional conflict scores for decision-aid users) and high acceptability ratings but mixed results for changes in knowledge. It should be noted that our web-based decision aid is designed for diverse populations mapped to these exact findings. Decision aids with a computer or web-based modality and tools that were tailored for underrepresented groups also showed positive effects on contraceptive use and decision self-efficacy [13], 2 outcomes that should be measured with future iterations of the tool. Our preliminary data on decisional conflict also shows promise for an effect on decisional quality.

We found no change in knowledge; the contraception systematic review found an increase in knowledge (up to 6 months) [13]. In our study population, a majority of patients’ contraceptive preferences also did not change before and after the intervention, which may indicate that patients had enough knowledge about their preferred option before seeking it from a clinician. We wondered whether there is a minimal amount of knowledge decision aids must have that is sufficient for quality contraceptive decision-making. Advisory panel clinicians discussed important information needed to make a decision, but we had to put constraints on how much content would be included in the tool to ensure a brief tool use time. Our results suggest that a focus on decisional conflict (helping patients feel more certain, more informed, have clearer values, and feel supported) may be a priority outcome, versus continuing to increase knowledge. Future work should define top priorities in contraceptive shared decision-making and illustrate these priorities’ effects on decision process outcomes (ie, decision quality—the quality of a decision made, regardless of the outcome).

### Limitations

The limitations of this study will guide future research on this decision aid. First, we only offered the decision aid in English and only recruited those who were fluent in English, thus reducing the generalizability of the tool to proficient English speakers and readers. Co-designing the tool to common languages spoken by racial or ethnic minorities (eg, Spanish, French, and Mandarin) would allow more patients to access the tool. Second, we did not capture a representative sample of the lesbian, gay, bisexual, transgender, queer community (6% of the study sample) in developing and evaluating the tool, potentially resulting in missing education pieces and decision-making factors for these individuals. We did recognize that patients with various gender identities and pronouns would be using the tool, resulting in the acquisition and addition of these data to the summary page for clinicians to easily access and acknowledge during clinical visits. Future work should make sure to represent stakeholders from this community within the development process.

Third, we only tested the tool at a Planned Parenthood clinic, which is not representative of all patients who could use the tool. This location provided a space to evaluate diverse populations, an important aspect of this study. Additionally, use of participants from ResearchMatch and similar clinical research volunteer banks are more likely to have access to a computer and the internet and may be more knowledgeable about scientific research than their peers. Future work should seek to co-design the tool with people that have lower levels of technology literacy and test the tool within various clinical settings in a randomized trial and be large enough to rigorously examine change by racial, ethnic, and gender identity groups. Finally, our pilot study required multiple manual steps to transfer decision aid summaries to clinicians, which could have caused disruptions to the clinical flow. To reduce disruptions and the risk of human error, future interventions should include an integrated approach such that a contraception tool securely exchanges information with the electronic health record. Patients and clinicians can then easily refer to the tool summary in subsequent visits.

### Conclusions

We developed and evaluated a web-based decision aid using patient-centered, shared decision-making, and health equity approaches to create a foundation of sexual health knowledge and aid patients with contraceptive method decision-making. Patients reported Healthy Sex Choices to be acceptable and experienced reductions in decisional conflict which is an improvement. This study laid a foundation for creating decision aids within reproductive health that acknowledge and provide guidance for diverse patient populations.

## Abbreviations

IPDAS: International Patient Decision Aid Standards
OHSU: Oregon Health & Science University
REDCap: research electronic data capture

## Data Availability

The data sets generated and analyzed during the study are not publicly available nor can be shared privately due to institutional review board protocol restrictions.
